# Using the Social Skills Improvement System (SSiS) Rating Scales to assess social skills in youth with Down syndrome

**DOI:** 10.3389/fpsyg.2023.1105520

**Published:** 2023-04-04

**Authors:** Marie Moore Channell, Laura J. Mattie, Emily K. Schworer, Deborah J. Fidler, Anna J. Esbensen

**Affiliations:** ^1^Department of Speech and Hearing Science, University of Illinois Urbana-Champaign, Champaign, IL, United States; ^2^Division of Developmental and Behavioral Pediatrics, Cincinnati Children’s Hospital Medical Center, Cincinnati, OH, United States; ^3^Waisman Center, University of Wisconsin-Madison, Madison, WI, United States; ^4^Department of Human Development and Family Studies, Colorado State University, Fort Collins, CO, United States; ^5^Univeristy of Cincinnati College of Medicine, Cincinnati, OH, United States

**Keywords:** social skills, down syndrome, challenging behavior, problem behaviors, social interaction, intellectual disability

## Abstract

**Introduction and Methods:**

This study provides preliminary data on the Social Skills Improvement System (SSiS) Rating Scales Parent Form to measure social skills in a sample of 124 children and adolescents with Down syndrome (DS) ages 6–17 years.

**Results:**

Overall, participants demonstrated relatively mild symptoms, with the sample’s average standard score falling within 1 standard deviation from the mean of the normative sample for the social skills (*M* = 92, *SD* = 15) and problem behaviors (*M* = 104, *SD* = 12) domains (normative sample *M* = 100, *SD* = 15 for both domains). However, a wide range of scores was observed across the sample for the composite and subscale scores. Differential patterns were also observed by subscale. For some subscales (i.e., Cooperation, Assertion, Responsibility, Engagement, Externalizing, Hyperactivity/Inattention, and Autism Spectrum), a disproportionate number of participants scored in the below average (i.e., lower levels of social skills) or above average (i.e., more symptomatic in problem behaviors or autism spectrum) range relative to the normative sample; for other subscales (i.e., Communication, Empathy, Self-Control, Bullying, and Internalizing), participants’ score distribution aligned more closely to that of the normative sample. SSiS composite scores correlated in the expected directions with standardized measures of autism characteristics, executive function, and expressive language.

**Discussion:**

This study provides some of the first evidence validating the use of the SSiS in youth with DS, filling a gap in standardized measures of social functioning in this population.

## 1. Introduction

Individuals with Down syndrome (DS) show a distinct yet complex phenotype that affects the language, cognitive, and social skills they use to interact and communicate with others (Iarocci et al., 2008; Cebula et al., 2010; Grieco et al., 2015; Thurman and del Hoyo Soriano, 2021). Research on social development in DS has focused mostly on early foundational skills, identifying several relative strengths (e.g., eye gaze, gestures, vocalizations, joint attention; Fidler, 2006; Fidler et al., 2008; Cebula et al., 2010; Thurman and del Hoyo Soriano, 2021). However, less is known about how individuals with DS use these foundational skills during social interactions in later childhood and adolescence. This lack of information represents a critical gap in the literature, given the range of individual differences in outcomes related to social development, such as independent living, employment, community participation, and quality of life, that have been reported among adults with DS (Iarocci et al., 2008; Scott et al., 2014; Jevne et al., 2022; Loveall et al., 2022). One major barrier is that few measures of social skills have been established in this population (Esbensen et al., 2017; Schworer et al., 2021). To address this barrier, the purpose of the current study was to evaluate the Social Skills Improvement System (SSiS) Rating Scales (Gresham and Elliott, 2008) for assessing social skills in a large sample of children and adolescents with DS.

Successful social interaction requires the coordination of many skills. For example, from the framework of social information processing theory (Crick and Dodge, 1994), an individual must show a general social orientation to pay attention to and encode the social cues around them. Then, the individual must use social cognition to interpret their social partner’s verbal and nonverbal communication and engage in social reasoning to make internal evaluations, ultimately deciding on a behavioral response. These processes also require the integration of other skills related to attention, emotion understanding, language processing, and emotion regulation (Lemerise and Arsenio, 2000; Grazzani et al., 2018).

Current research on key social functioning skills in DS indicates that children and adolescents often show floor effects or low performances on measures of social cognition that involve tasks like perspective-taking, social reasoning, and social problem-solving (Abbeduto et al., 2008; Hahn et al., 2013; Ashby et al., 2017; Barisnikov and Lejeune, 2018; Martin et al., 2018; Schworer et al., 2021). However, these tasks tend to rely heavily on language processing and executive function, known areas of difficulty in DS, posing a challenge for assessing social cognition *per se* (see Channell and Loveall, 2021). Similarly, youth with DS show difficulties in aspects of pragmatic language, again using tasks that require higher-order social cognition (i.e., perspective-taking or theory of mind) and language (Lee et al., 2017; Smith et al., 2017). If individuals with DS demonstrate lower performances on these tasks in experimental settings, it is possible this translates to difficulties during day-to-day social interactions, especially as they progress into adolescence when social demands increase (Iarocci et al., 2008). However, it is also possible that in more naturalistic settings, individuals with DS may benefit from situational and nonverbal cues (e.g., gesturing, eye gaze, emotion recognition) to help them navigate social interactions. For this reason, more ecologically valid assessments are needed.

In clinical settings, a common approach for assessing social skills in childhood and adolescence is through informant report (e.g., caregiver, teacher, self, peers). Informant report measures are particularly helpful for understanding social behavior in naturalistic settings and across different environments (e.g., home, school, peer interactions). One such measure developed for this purpose is the SSiS (Gresham and Elliott, 2008), which is the newer version of the Social Skills Rating System (SSRS; Gresham and Elliott, 1990). The SSRS and SSiS were developed for clinical use to identify children who have delays in social skills and challenging behaviors that affect social interaction to develop intervention targets. To our knowledge, only two studies have examined the SSRS in small samples of 4- to 6-year-olds with DS through parent (Guralnick et al., 2009) and teacher (Guralnick et al., 2011) report. In both studies, young children with DS had lower social skills standard scores than both chronological and mental age-matched neurotypical peers, suggesting that even in early childhood, this measure is sensitive to delays in social skills in DS. These findings suggest that even in early childhood, children with DS may show a profile of difficulties in some areas of social development and in related domains that are also critical to social interaction [i.e., expressive language, internalizing and externalizing problems, hyperactivity, and distractibility (Guralnick et al., 2009, 2011)]. To our knowledge, no study to date has systematically measured social skills across older children and adolescents with DS. Such information is needed to understand social skills in DS within the framework of the social demands experienced by older youth.

A few studies have used other caregiver report measures of social challenges that were originally developed to capture autism symptoms [i.e., Social Responsiveness Scale (SRS; Constantino and Gruber, 2005); Social Responsiveness Scale-2nd edition (SRS-2; Constantino and Gruber, 2012); Children’s Social Behavior Questionnaire (Hartman et al., 2007)] to examine social skills in youth with DS. Across these studies, children and adolescents with DS tend to score in the elevated range, showing more social challenges relative to chronological age- and sex-based norms from the general population (van Gameren-Oosterom et al., 2013; Channell et al., 2015; Channell, 2020; Schworer et al., 2021). Although these measures were designed to capture challenges in social communication and interaction in the context of autism risk, they show potential as measures of broader social outcomes for individuals with DS (Schworer et al., 2021). However, more research is needed to support their use in this population and to systematically examine profiles of social skills in youth with DS. The current study seeks to address the latter.

In addition to their clinical use, measures of social skills can contribute to the understanding of the DS phenotype. Over the past few decades, a robust body of research has characterized the behavioral phenotype associated with DS in different developmental domains (see Grieco et al., 2015; Thurman and del Hoyo Soriano, 2021 for reviews). For example, individuals with DS tend to show relative difficulties in the domains of expressive language (Abbeduto et al., 2007; McDuffie et al., 2017), motor development (Winders, 2013; Frank and Esbensen, 2015), and auditory processing (Conners et al., 2011), with relative strengths in aspects of visuospatial processing (Yang et al., 2014) and early social skill development (Fidler et al., 2008). This profile begins to emerge early in life and builds over time as children with DS adapt and develop strategies to interact with the world around them (Fidler, 2005; D’Souza and D’Souza, 2022). Because development in one domain affects subsequent development in related domains, unique intra-individual profiles continue to evolve across childhood (D’souza et al., 2017; Fidler et al., 2019; D’Souza and D’Souza, 2022). Furthermore, these developmental profiles affect, and are affected by, the individual’s environment and neurogenetics, resulting in considerable inter-individual differences in developmental trajectories (Cebula et al., 2010; Karmiloff-Smith et al., 2016). By examining social skill profiles in children and adolescents with DS alongside related domains such as expressive language, executive function, and autism characteristics, we can gain insight into their interrelatedness and advance the understanding of the distinct, yet complex social behavioral phenotype associated with DS.

The purpose of this study was to provide preliminary data on the SSiS to measure social skills in a large sample of children and adolescents with DS. To accomplish this, the aims were to: (1) describe social skills and related behaviors captured by the SSiS in youth with DS; and (2) examine associations between the SSiS and measures of related domains (i.e., autism characteristics, executive function, and expressive language as measured by vocabulary). We expected that social skills measured by the SSiS would be negatively associated with autism characteristics and executive function impairments and positively associated with expressive language. We expected that behavioral problems measured by the SSiS would be positively associated with autism characteristics and executive function impairments and negatively associated with expressive language.

## 2. Materials and methods

### 2.1. Participants

Participants were combined from two research studies—a study on language in DS at the University of Illinois Urbana-Champaign (UIUC) and a multi-site study on measuring cognitive constructs at Cincinnati Children’s Hospital Medical Center (CCHMC) and Colorado State University (CSU). Both studies were approved by the respective institution’s Institutional Review Board. Eligibility criteria for the UIUC study were that the child with DS was between 6 and 11 years old, spoke English as a native language, communicated primarily through speech, and was able to speak in at least 2- to 3-word phrases according to parent report. Eligibility criteria for the CCHMC/CSU study were that the child with DS was between 6 and 17 years old, had English spoken as the primary language at home, and had a parent-reported developmental age of at least 3 years in order to engage in neuropsychological testing. No children were excluded from participation at CCHMC/CSU based on parental report of developmental age.

To be included in the current analyses, participants were required to have complete data on the SSiS. Thirteen participants from CCHMC/CSU were excluded due to missing SSiS data. This resulted in a sample size of 124 (*M* age = 11.61, *SD* = 3.48), *n* = 40 from UIUC and *n* = 84 from CCHMC/CSU. See Table 1 for demographic characteristics of the sample.

**Table 1:** Participant demographic characteristics.

	Full sample *n* = 124	UIUC site *n* = 40	CCHMC/CSU site *n* = 84
Age in years	*M* = 11.61 (*SD* = 3.48)^*^	*M* = 8.55 (*SD* = 1.62)	*M* = 13.07 (*SD* = 3.17)
**Sex**
Male	47.6% (*n* = 59)	35.0% (*n* = 14)	53.6% (*n* = 45)
Female	52.4% (*n* = 65)	65.0% (*n* = 26)	46.4% (*n* = 39)
**Race and ethnicity**
White	83.9% (*n* = 104)	77.5% (*n* = 31)	86.9% (*n* = 73)
Black	8.1% (*n* = 10)	12.5% (*n* = 5)	6.0% (*n* = 5)
Asian	4.0% (*n* = 5)	0	6.0% (*n* = 5)
Other	3.2% (*n* = 4)	7.5% (*n* = 3)	1.2% (*n* = 1)
Unreported	0.8% (*n* = 1)	2.5% (*n* = 1)	0
Hispanic	5.6% (*n* = 7)	2.5% (*n* = 1)	7.1% (*n* = 6)
Non-Hispanic	93.5% (*n* = 116)	97.5% (*n* = 39)	91.7% (*n* = 77)
Unreported	0.8% (*n* = 1)	0	1.2% (*n* = 1)

* Independent samples *t*-test indicated statistically significant differences between sites (*p* < 0.01).

### 2.2. Study design

As part of the larger studies, caregivers completed a series of questionnaires, including the SSiS (Gresham and Elliott, 2008), SRS-2 (Constantino and Gruber, 2012), and the Behavior Rating Inventory of Executive Function, 2nd edition (BRIEF2; Gioia et al., 2015). The children with DS also completed a direct assessment battery for each study that included the Expressive Vocabulary Test, 2^nd^ or 3^rd^ edition (EVT-2; Williams, 2007; EVT-3; Williams, 2019) and an IQ test. All UIUC participants were administered the EVT-2 (*n* = 37 after excluding 3 participants due to examiner error); CCHMC/CSU participants were administered the EVT-2 (*n* = 12) or the EVT-3 (*n* = 72), depending on year of enrollment. As a descriptive measure of IQ, UIUC participants completed the Leiter International Performance Test, 3^rd^ edition (Leiter-3; Roid and Miller, 2013) nonverbal IQ test, and CCHMC/CSU participants completed the abbreviated version of the Stanford-Binet Intelligence Scales, 5th edition (SB-5; Roid, 2003; See Table 2).

**Table 2:** Participant performance on study measures.

	Full sample *M (SD) range*	UIUC site *M (SD) range*	CCHMC/CSU site *M (SD) range*
SSiS Social Skills composite	91.94 (14.62) 49–123	94.25 (13.22) 58–123	90.85 (15.19) 49–123
SSiS Problem Behaviors composite	104.39 (12.06)^*^ 82–136	110.28 (12.09) 84–136	101.58 (11.05) 82–136
SRS-2 total *T*-score^a^	59.96 (8.66) 42–86	61.38 (8.09) 44–83	59.26 (8.89) 42–86
SRS-2 Restricted Interests and Repetitive Behaviors *T*-score^a^	60.46 (11.15)^*^ 43–96	64.70 (11.38) 44–96	58.37 (10.49) 43–90
SRS-2 Social Communication and Interaction *T*-score^a^	59.55 (8.33) 42–84	60.13 (7.71) 44–81	59.27 (8.66) 42–84
BRIEF2 Global Executive Composite^b^	59.65 (8.95)^*^ 38–81	63.79 (8.00) 48–80	57.77 (8.77) 38–81
BRIEF2 Behavioral Regulation Index	58.76 (9.32)^*^ 37–82	62.63 (8.33) 46–82	56.92 (9.25) 37–76
BRIEF2 Emotional Regulation Index	55.71 (10.08)^*^ 39–82	60.43 (10.61) 40–82	53.46 (9.05) 39–79
BRIEF2 Cognitive Regulation Index^a^	59.85 (8.52)^*^ 38–84	63.03 (7.48) 45–77	58.42 (8.61) 38–84
EVT-2/3 standard score^a^^,^^c^	62.91 (12.98) 20–94	64.68 (12.97) 41–94	62.13 (12.98) 20–89
Leiter-3 nonverbal IQ	–	59.13 (9.49) 36–75	–
SB-5 ABIQ	–	–	49.36 (5.61) 47–76

* Independent samples t-test indicated statistically significant differences between sites (*p* < 0.01).

a *n* = 121;

b *n* = 122;

c EVT-2: *n* = 37 UIUC and *n* = 12 CCHMC/CSU, EVT-3: *n* = 72 CCHMC/CSU.

SSiS = Social Skills Improvement System. SRS-2 = Social Responsiveness Scale, 2nd edition. BRIEF2 = Behavior Rating Inventory of Executive Function, 2nd edition. EVT-2/3 = Expressive Vocabulary Test, 2nd edition/3rd edition. Leiter-3 = Leiter International Performance Scale, 3rd edition. SB-5 ABIQ = Stanford-Binet Intelligence Scales, 5th edition, Abbreviated Battery Intelligence Quotient.

### 2.3. Measures

#### 2.3.1. Social skills

The SSiS Parent Form (Gresham and Elliott, 2008) is a standardized, norm-referenced questionnaire that asks caregivers to rate their child’s behaviors as they relate to everyday social interactions. The SSiS informs intervention by identifying a child’s social skills strengths and difficulties in acquisition or performance, including the presence of challenging behaviors that affect social interaction. Caregivers rate the frequency of each social skill (46 items) or problem behavior (33 items) over the last 2 months as *Never*, *Seldom*, *Often*, or *Almost Always*. For the social skills items, caregivers also rate the perceived importance of the behavior for their child’s development (*not important*, *important*, or *critical*), but these ratings are not factored into the composite scores.

The Social Skills subscales are *Communication* (pragmatic skills), *Cooperation* (helping others, sharing, and compliance), *Assertion* (requesting, initiating, and responding appropriately), *Responsibility* (showing respect of property and communicating with adults), *Empathy* (showing concern for others’ feelings and perspectives), *Engagement* (joining and inviting others to join activities, making friends), and *Self-Control* (responding appropriately to conflict and compromising). The Problem Behaviors subscales are *Externalizing* (verbal or physical aggression), *Bullying* (hurting others physically or emotionally, excluding others), *Hyperactivity/Inattention* (fidgety, impulsive, and easily distracted), and *Internalizing* (anxious, sad, or lonely). There is also an *Autism Spectrum* subscale (difficulty connecting with others, repetitive behaviors, and rigidity) composed of items that span across the Social Skills and Problem Behaviors domains.

Subscale scores were converted to categorical ‘Behavioral Levels’ based on the raw score distribution in the normative sample. ‘Below Average’ indicates scores that are more than 1 SD below the normative sample mean, ‘Average’ indicates scores within ± 1 SD from the mean, and ‘Above Average’ indicates scores that are more than 1 SD above the mean. Composite scores for the two domains, Social Skills and Problem Behaviors, were computed based on chronological age and sex. Composite scores have a mean of 100 and standard deviation of 15. For all score types, higher scores in the Social Skills domain indicate stronger skills, and higher scores in the Problem Behaviors domain indicate more impairment. The SSiS publishers report high internal consistency for the Parent Form (median reliability *α* = 0.94–0.96 for composite scales; median reliability *α* = 0.83–0.87 for subscales) for the age ranges represented in the current study. They also reported moderate to strong correlations with the Behavior Assessment System for Children, 2nd edition (BASC-2; Reynolds and Kamphaus, 2008) for the age ranges represented in the current study (SSiS Social Skills and BASC-2 Adaptive Skills *r* = 0.62–0.66; SSiS Problem Behaviors and BASC-2 Behavioral Symptoms Index *r* = 0.80–0.82). Reported test–retest reliability for the SSiS Parent Form is also strong (median *r* = 0.87 for composite scales; median *r* = 0.83 for subscales).

#### 2.3.2. Autism characteristics

The SRS-2 School-Age Form (Constantino and Gruber, 2012) is a 65-item standardized caregiver-report autism symptom screener for children ages 2.5–18 years. Caregivers rate the presence and frequency of their child’s behaviors within the last 6 months. The SRS-2 has two domains that align with DSM-5 diagnostic criteria for autism spectrum disorder—Social Communication and Interaction (SCI; with subdomains of *Social Awareness*, *Social Cognition*, *Social Communication*, and *Social Motivation*) and Restricted, Repetitive Behaviors and Interests (RRB). Chronological age and sex normed *T*-scores (*M* = 50, *SD* = 10) were computed for the SCI and RRB; an overall T-score was also computed. Higher scores indicate more autism-like symptoms. The SRS-2 publishers report strong psychometric properties in the standardization sample of children with and without autism for the School-Age Form (internal consistency *α* = 0.95–97). Strong psychometric properties have also been reported for the SRS-2 in a smaller sample of children and adolescents with DS (Schworer et al., 2021).

#### 2.3.3. Executive functioning

The BRIEF2 School-Age (Gioia et al., 2015) is a 63-item standardized caregiver-report questionnaire of everyday executive functioning for children ages 5–18 years. Caregivers rate the frequency in which their child engages in different behaviors in three categories that span different domains. The Behavioral Regulation Index (BRI) includes the domains of *Inhibit* and *Self-Monitor*. The Emotional Regulation Index (ERI) includes the domains of *Shift* and *Emotional Control*. The Cognitive Regulation Index (CRI) includes the domains of *Initiate*, *Working Memory*, *Plan/Organize*, *Task-Monitor*, and *Organization of Materials*.

T-scores were computed for each domain score and for the BRI, ERI, and CRI based on chronological age and sex norms. A Global Executive Composite (GEC) was also computed from chronological age and sex. Domain, index, and composite T-scores are all on the same scale (*M* = 50, *SD* = 10); higher scores indicate more dysregulation of executive function. The BRIEF2 publishers report strong test–retest reliability (*r* = 0.88 for the GEC; *r* = 0.82–0.89 for the index scores) and high internal consistency reliability (*α* = 0.97 for the GEC; *α* = 0.90–0.96 for the index scores) for the Parent Form.

#### 2.3.4. Expressive language: Vocabulary

The EVT-2 (Williams, 2007) and EVT-3 (Williams, 2019) are different editions of a standardized measure of expressive vocabulary normed for ages 2.5 – 90 + years. For both versions, examinees are shown a series of pictures and are asked to verbally label each picture. Specific item content and basal/ceiling rules were updated in the EVT-3. The version that participants were administered depended on the timing of their study entry. Age-normed standard scores (*M* = 100, *SD* = 15) were used in data analysis. The EVT-2 and EVT-3 publishers report strong test–retest reliability (0.95 and 0.88, respectively). The versions are also highly correlated (*r* = 0.86; Williams, 2019).

#### 2.3.5. Cognition

The Leiter-3 (Roid and Miller, 2013) is a standardized test of nonverbal cognition normed for ages 3 – 75 + years. It is nonverbal in administration and in method of response; examiners use gestures and facial expressions to model instructions, and examinees use pointing and other gestures to indicate their response. All UIUC participants completed the four Leiter-3 subtests (*Figure Ground*, *Form Completion*, *Classification and Analogies*, and *Sequential Order*) that yield a nonverbal IQ composite score (*M* = 100, *SD* = 15). The Leiter-3 publishers report good internal consistency reliability across composite scores (0.94–0.98) and its validation against the nonverbal IQ portion of the Stanford-Binet, 5th edition (*r* = 0.77). The SB-5 (Roid, 2003) is a standardized test of cognition that includes both verbal and nonverbal ability and is normed for ages 2 – 85 + years. All CCHMC/CSU participants completed the abbreviated battery IQ (ABIQ).

### 2.4. Data analysis plan

To address Aim 1, we first examined the distribution of SSiS Social Skills and Problem Behaviors composite scores across the sample. Next, we examined the distribution of subscale raw scores by behavioral level (Below Average, Average, or Above Average). For Aim 2, we conducted correlational analyses between the SSiS and the SRS-2, BRIEF2, and EVT-2/3. For these analyses, we included the SSiS subscales to determine the extent to which the different subscales demonstrate shared vs. distinct characteristics. Missing data (*n* = 3 EVT-2 from UIUC; *n* = 3 SRS-2 from CCHMC/CSU; *n* = 2 BRIEF2 CRI and GEC from CCHMC/CSU) were excluded pairwise such that the cases were excluded only from the correlational analyses involving the missing variables.

## 3. Results

### 3.1. Aim 1: Describe social skills and related behaviors captured by the SSiS in youth with DS

**Figure 1:**
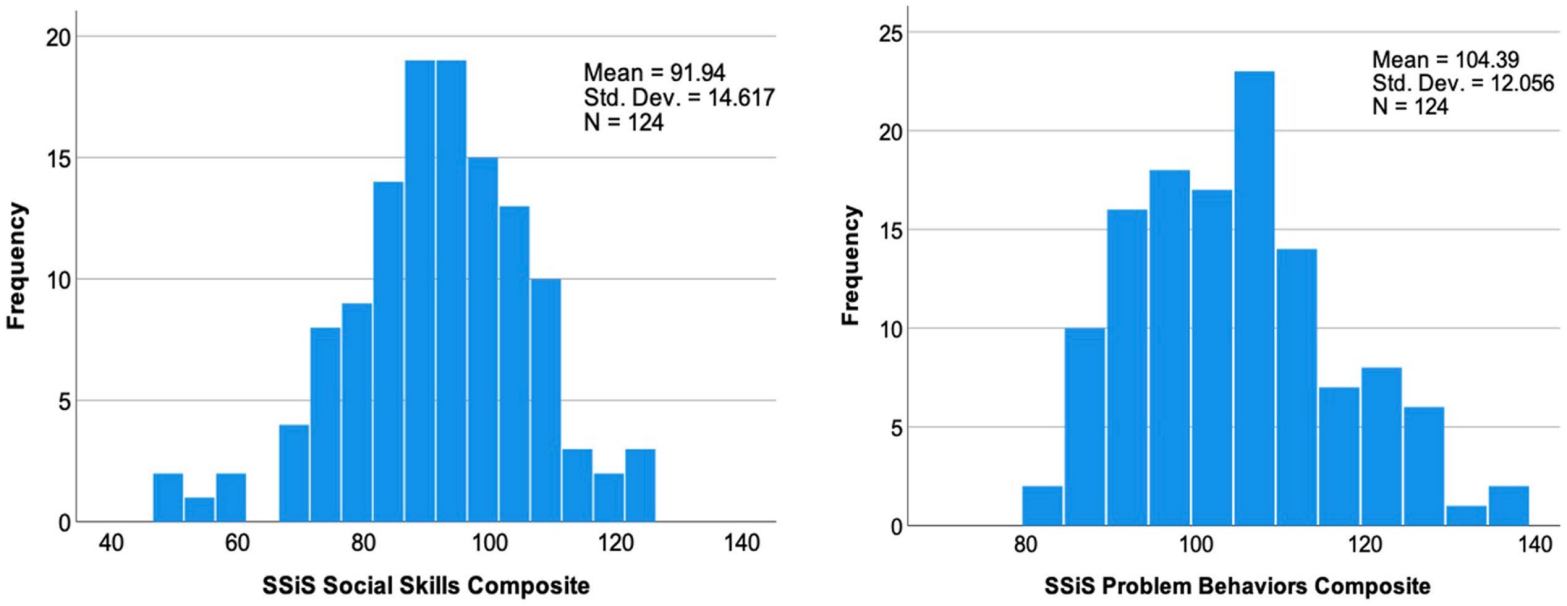
Distribution of Social Skills Improvement System (SSiS) composite scores.

Table 2 provides the SSiS Social Skills and Problem Behaviors composite scores for the sample; Figure 1 shows the distribution of these scores. For Social Skills, skewness was −0.44 and kurtosis was 0.66. For Problem Behaviors, skewness was 0.45 and kurtosis was −0.29.

**Figure 2:**
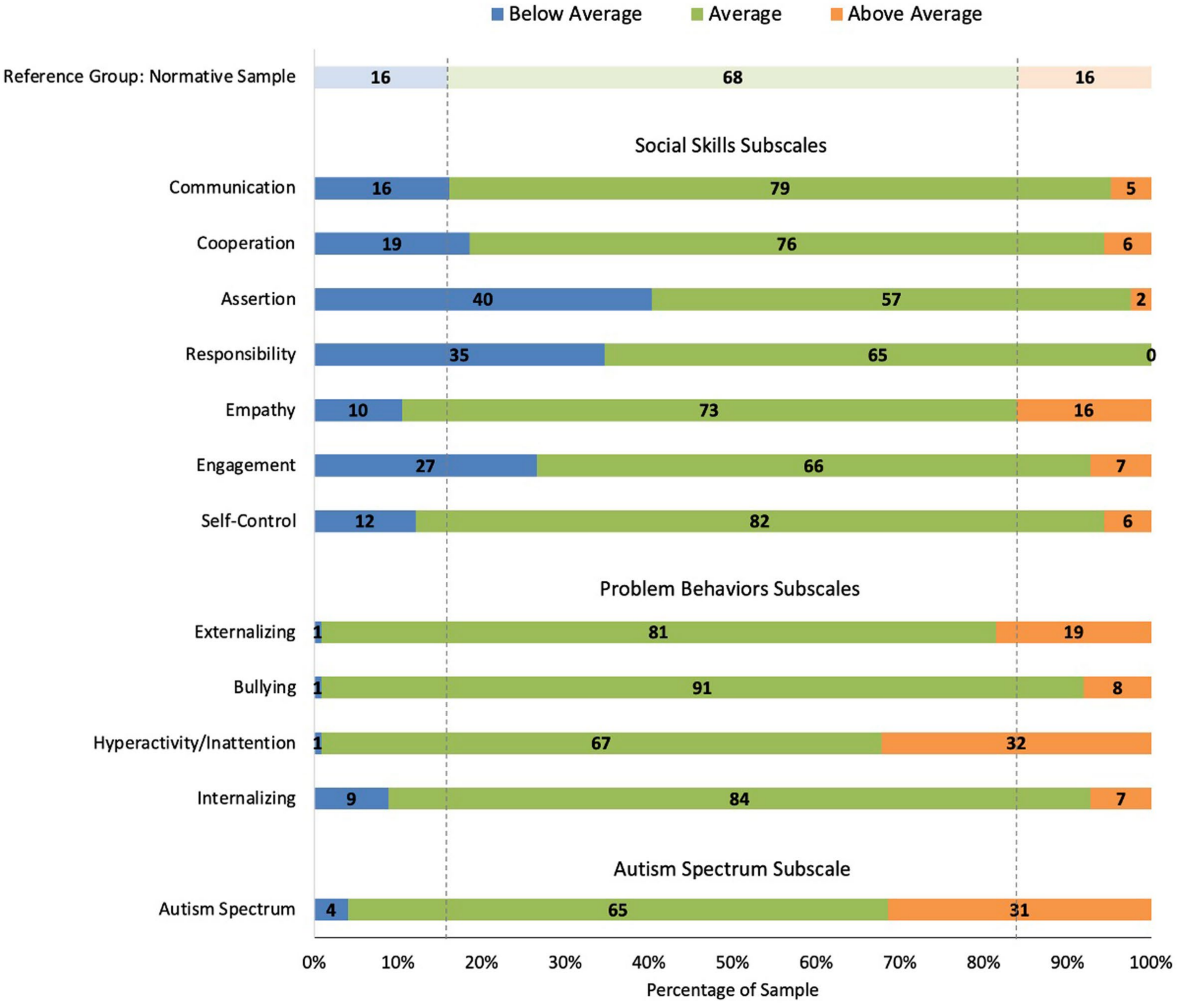
Percent of people with Down syndrome (DS) in each behavioral level category for the SSiS subscales.

Figure 2 provides the distribution of subscale raw scores across the behavioral level categories (i.e., Below Average, Average, or Above Average), with the normative sample shown as a reference group. In the SSiS normative sample, the behavioral level categories were built around the raw score distributions for each subscale such that ‘Below/Above Average’ indicates ≥ 1 SD from the mean. Thus, 16% of the normative sample had scores that fell in the Below/Above Average categories. In the current sample of children with DS, the Social Skills subscales for which *more than* 16% scored in the Below Average category (i.e., less developed skills) were Cooperation (19%, *n* = 23), Assertion (40%, *n* = 50), Responsibility (35%, *n* = 43), and Engagement (27%, *n* = 33). The Problem Behaviors subscales for which *more than* 16% of the sample scored in the Above Average category (i.e., more challenging behaviors or dysregulation) were Externalizing (19%, *n* = 23) and Hyperactivity/Inattention (32%, *n* = 40). Finally, a relatively large portion of children scored in the Above Average (i.e., more symptomatic) category for the Autism Spectrum subscale (31%, *n* = 39).

### 3.2. Aim 2: Examine associations between the SSiS and measures of related domains

We conducted Pearson’s *r* correlational analyses between SSiS composite standard scores, participant age, and composite/index/standard scores from the SRS-2, BRIEF2, and EVT-2/3. Correlations between these measures and SSiS Social Skills and Problem Behaviors are provided in Table 3. Overall, the SSiS Social Skills composite demonstrated large negative correlations with SRS-2 T-scores, moderate negative correlations with BRIEF2 Index scores, and a moderate positive correlation with EVT-2/3 standard scores. The SSiS Problem Behaviors composite showed large positive correlations with SRS-2 T-scores and BRIEF2 Index scores but was not significantly correlated with EVT-2/3 standard scores. Neither SSiS Social Skills nor Problem Behaviors composites were significantly correlated with participant age.

**Table 3:** Pearson’s *r* correlation coefficients for SSiS composite scores and other study measures.

	Age	SSiS SS	SSiS PB	SRS-2 total	SRS-2 RRB	SRS-2 SCI	BRIEF2 GEC	BRIEF2 BRI	BRIEF2 ERI	BRIEF2 CRI	EVT-2/3
Age	1	−0.040	−0.075	0.053	−0.024	0.069	−0.165	−0.178^*^	−0.088	−0.106	−0.177
SSiS Social Skills (SS)	–	1	−0.413^**^	−0.690^**^	−0.472^**^	−0.719^**^	−0.274^**^	−0.289^**^	−0.252^**^	−0.226^*^	0.359^**^
SSiS Problem Behaviors (PB)	–	–	1	0.631^**^	0.663^**^	0.572^**^	0.700^**^	0.604^**^	0.692^**^	0.574^**^	−0.107
SRS-2 total *T*-score	–	–	–	1	0.829^**^	0.977^**^	0.499^**^	0.452^**^	0.507^**^	0.408^**^	−0.218^*^
SRS-2 RRB *T*-score	–	–	–	–	1	0.696^**^	0.572^**^	0.536^**^	0.639^**^	0.424^**^	−0.253^**^
SRS-2 SCI *T*-score	–	–	–	–	–	1	0.435^**^	0.393^**^	0.421^**^	0.369^**^	−0.195^*^
BRIEF2 GEC	–	–	–	–	–	–	1	0.848^**^	0.789^**^	0.913^**^	−0.098
BRIEF2 BRI	–	–	–	–	–	–	–	1	0.635^**^	0.693^**^	−0.176
BRIEF2 ERI	–	–	–	–	–	–	–	–	1	0.580^**^	−0.138
BRIEF2 CRI	–	–	–	–	–	–	–	–	–	1	−0.034
EVT-2/3 standard score	–	–	–	–	–	–	–	–	–	–	1

^*^p < 0.05, ^**^p < 0.01.

The shaded cells indicate significance at *p* < 0.05. SSiS = Social Skills Improvement System. SRS-2 = Social Responsiveness Scale, 2nd edition. RRB = Restricted, Repetitive Behaviors and Interests. SCI = Social Communication and Interaction. BRIEF2 = Behavior Rating Inventory of Executive Function, 2nd edition. GEC = Global Executive Composite. BRI = Behavior Regulation Index. ERI = Emotion Regulation Index. CRI = Cognitive Regulation Index. EVT-2/3 = Expressive Vocabulary Test, 2nd edition/3rd edition.

Correlations between the SSiS subscale raw scores and other measures are reported in Tables 4–6. Differential patterns emerged such that all the Social Skills subscales were significantly negatively correlated with SRS-2 scores and significantly positively correlated with EVT-2/3 scores; however, only some subscales were significantly correlated with different index scores of the BRIEF2 (Table 4), demonstrating differentiation across subdomains.

**Table 4:** Pearson’s *r* correlation coefficients for SSiS Social Skills subscale raw scores and other study measures.

	Age	SSiS Comm	SSiS Coop	SSiS Assert	SSiS Resp	SSiS Emp	SSiS Engage	SSiS S-C	SRS-2 total T	SRS-2 RRB	SRS-2 SCI	BRIEF2 GEC	BRIEF2 BRI	BRIEF2 ERI	BRIEF2 CRI	EVT-2/3
Age	1	−0.134	0.133	−0.124	0.192^*^	0.009	−0.111	0.093	0.053	−0.024	0.069	−0.165	−0.178^*^	−0.088	−0.106	−0.177
SSiS Communication (Comm)		1	0.539^**^	0.636^**^	0.582^**^	0.659^**^	0.585^**^	0.518^**^	−0.566^**^	−0.328^**^	−0.611^**^	−0.187^*^	−0.183^*^	−0.133	−0.126	0.296^**^
SSiS Cooperation (Coop)			1	0.343^**^	0.831^**^	0.451^**^	0.346^**^	0.562^**^	−0.489^**^	−0.433^**^	−0.476^**^	−0.414^**^	−0.434^**^	−0.334^**^	−0.349^**^	0.258^**^
SSiS Assertion (Assert)				1	0.438^**^	0.483^**^	0.548^**^	0.419^**^	−0.537^**^	−0.279^**^	−0.589^**^	−0.018	−0.075	−0.036	0.012	0.288^**^
SSiS Responsibility (Resp)					1	0.525^**^	0.368^**^	0.583^**^	−0.528^**^	−0.425^**^	−0.531^**^	−0.359^**^	−0.408^**^	−0.296^**^	−0.272^**^	0.326^**^
SSiS Empathy (Emp)						1	0.571^**^	0.405^**^	−0.500^**^	−0.351^**^	−0.521^**^	−0.162	−0.168	−0.170	−0.079	0.248^**^
SSiS Engagement (Engage)							1	0.391^**^	−0.583^**^	−0.387^**^	−0.605^**^	−0.164	−0.094	−0.156	−0.187^*^	0.272^**^
SSiS Self-Control (S-C)								1	−0.443^**^	−0.331^**^	−0.456^**^	−0.297^**^	−0.307^**^	−0.380^**^	−0.182^*^	0.234^**^

^*^p < 0.05, ^**^p < 0.01.

The shaded cells indicate significance at *p* < 0.05. SSiS = Social Skills Improvement System. SRS-2 = Social Responsiveness Scale, 2nd edition. RRB = Restricted, Repetitive Behaviors and Interests. SCI = Social Communication and Interaction. BRIEF2 = Behavior Rating Inventory of Executive Function, 2nd edition. GEC = Global Executive Composite. BRI = Behavior Regulation Index. ERI = Emotion Regulation Index. CRI = Cognitive Regulation Index. EVT-2/3 = Expressive Vocabulary Test, 2nd edition/3rd edition.

**Table 5:** Pearson’s *r* correlation coefficients for SSiS Problem Behaviors subscale raw scores and other study measures.

	Age	SSiS Extern	SSiS Bully	SSiS Hyp/Inatt	SSiS Intern	SRS-2 total T	SRS-2 RRB	SRS-2 SCI	BRIEF2 GEC	BRIEF2 BRI	BRIEF2 ERI	BRIEF2 CRI	EVT-2/3
Age	1	−0.313^**^	−0.139	−0.321^**^	0.091	0.053	−0.024	0.069	−0.165	−0.178^*^	−0.088	−0.106	−0.177
SSiS Externalizing (Ext)		1	0.653^**^	0.855^**^	0.458^**^	0.425^**^	0.502^**^	0.369^**^	0.678^**^	0.642^**^	0.616^**^	0.517^**^	−0.011
SSiS Bullying (Bully)			1	0.463^**^	0.294^**^	0.262^**^	0.347^**^	0.219^*^	0.512^**^	0.460^**^	0.421^**^	0.353^**^	−0.083
SSiS Hyperactivity/Inattention (Hyp/Inatt)				1	0.423^**^	0.507^**^	0.579^**^	0.448^**^	0.693^**^	0.646^**^	0.602^**^	0.556^**^	−0.019
SSiS Internalizing (Intern)					1	0.529^**^	0.420^**^	0.521^**^	0.398^**^	0.232^**^	0.463^**^	0.378^**^	0.015

^*^p < 0.05, ^**^p < 0.01.

The shaded cells indicate significance at *p* < 0.05. SSiS = Social Skills Improvement System. SRS-2 = Social Responsiveness Scale, 2nd edition. RRB = Restricted, Repetitive Behaviors and Interests. SCI = Social Communication and Interaction. BRIEF2 = Behavior Rating Inventory of Executive Function, 2nd edition. GEC = Global Executive Composite. BRI = Behavior Regulation Index. ERI = Emotion Regulation Index. CRI = Cognitive Regulation Index. EVT-2/3 = Expressive Vocabulary Test, 2nd edition/3rd edition.

**Table 6:** Pearson’s *r* correlation coefficients for SSiS autism spectrum subscale raw scores and other study measures.

	Age	SSiS Autism	SRS-2 total T	SRS-2 RRB	SRS-2 SCI	BRIEF2 GEC	BRIEF2 BRI	BRIEF2 ERI	BRIEF2 CRI	EVT-2/3
Age	1	0.002	0.053	−0.024	0.069	−0.165	−0.178^*^	−0.088	−0.106	−0.177
SSiS Autism Spectrum		1	0.791^**^	0.704^**^	0.764^**^	0.451^**^	0.365^**^	0.507^**^	0.314^**^	−0.290^**^

^*^p < 0.05, ^**^p < 0.01.

The shaded cells indicate significance at *p* < 0.05. SSiS = Social Skills Improvement System. SRS-2 = Social Responsiveness Scale, 2nd edition. RRB = Restricted, Repetitive Behaviors and Interests. SCI = Social Communication and Interaction. BRIEF2 = Behavior Rating Inventory of Executive Function, 2nd edition. GEC = Global Executive Composite. BRI = Behavior Regulation Index. ERI = Emotion Regulation Index. CRI = Cognitive Regulation Index. EVT-2/3 = Expressive Vocabulary Test, 2nd edition/3rd edition.

In contrast, all the Problem Behaviors subscales showed significant positive correlations with all BRIEF2 index scores, though strength of the correlations varied across subscales (Table 5). The Problem Behaviors subscales were also significantly positively correlated with SRS-2 scores, but no subscales were significantly correlated with EVT-2/3 scores. Finally, the Autism Spectrum subscale showed significant positive correlations across all SRS-2 and BRIEF2 scores as well as a significant negative correlation with EVT-2/3 scores (Table 6).

### 3.3. *Post hoc* analyses: Deeper characterization of the sample by SSiS behavioral level

Because a disproportionate number of children fell in the Below/Above Average behavioral level categories (i.e., compared to the normative sample) across several subscales, we conducted additional *post hoc* analyses to better characterize those participants. The goal was to subdivide the sample based on the SSiS behavioral levels and then examine patterns of characteristics across related domains (i.e., autism characteristics, executive function, and expressive language as measured by vocabulary). For these analyses, we focused only on the SSiS subscales for which more than 16% of the sample scored in the Below Average range for the Social Skills subscales or the Above Average range for the Problem Behaviors subscales. We converted composite/index/standard scores from the SRS-2, BRIEF2, and EVT-2/3 to *Z*-scores and plotted mean *Z*-scores for the SSiS Below/Above Average group relative to the rest of the participant sample.

**Figure 3:**
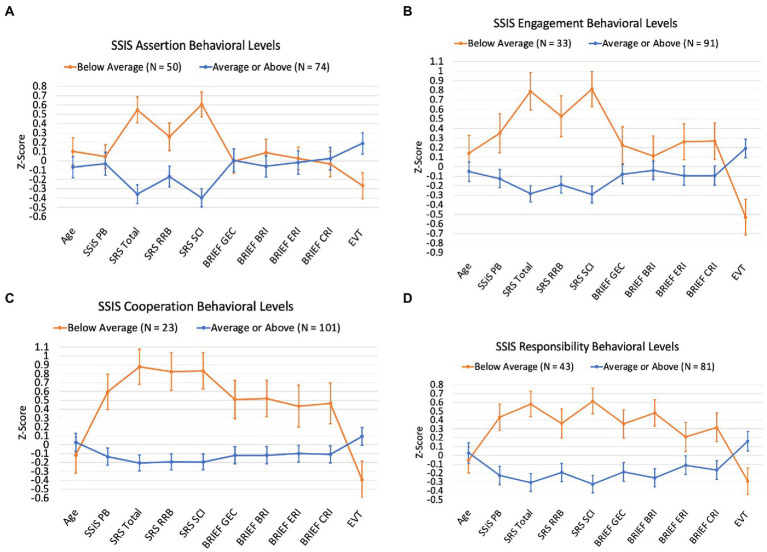
Participant scores across study measures by SSiS Social Skills subscale behavioral levels.

Figure 3 shows performance across other study measures for the group of children with DS whose SSiS Social Skills subscale scores fell in the Below Average category relative to the remainder of the participant sample (i.e., those whose subscale scores fell in the Average or Above Average categories). For Assertion, the two subgroups diverged such that the ‘Below Average’ group had more elevated SRS-2 scores and lower EVT-2/3 scores. For Engagement, Cooperation, and Responsibility, the two subgroups diverged on all study measures. Overall, the participants in the ‘Below Average’ group for these three subscales had higher SSiS Problem Behaviors and higher (i.e., more symptomatic) SRS-2 and BRIEF2 scores. They also had lower EVT-2/3 scores. Finally, the participants in the ‘Below Average’ group for Engagement were older in age.

**Figure 4:**
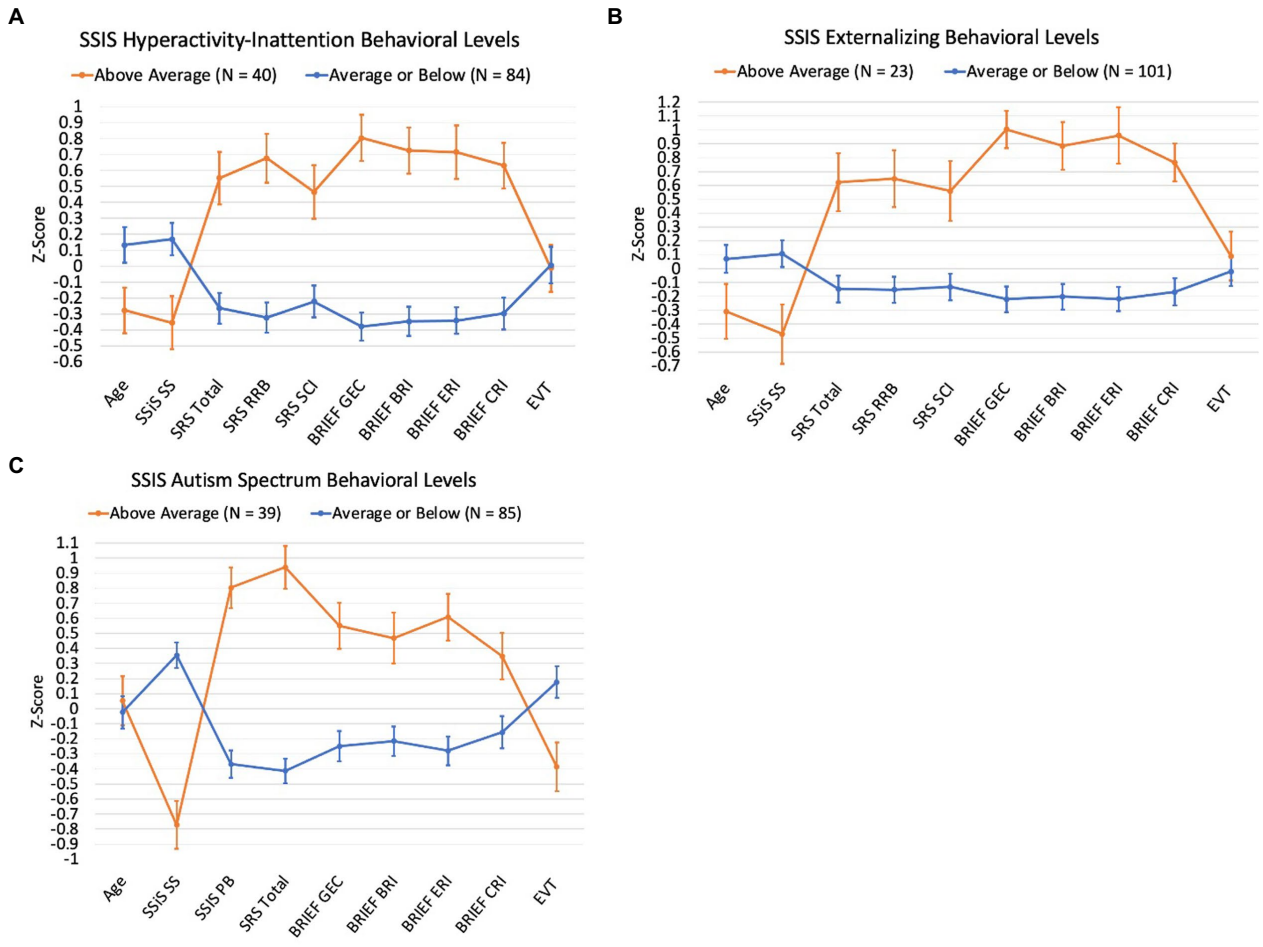
Participant scores across study measures by SSiS Problem Behaviors and Autism Spectrum subscale behavioral levels

Figure 4 shows performance across other study measures for the group of children with DS whose SSiS Problem Behaviors and Autism Spectrum subscale scores fell in the Above Average category relative to the remainder of the participant sample (i.e., those whose subscale scores fell in the Average or Below Average categories). For Hyperactivity/Inattention, Externalizing, and Autism Spectrum subscales, the ‘Above Average’ groups showed lower SSiS Social Skills scores and higher (i.e., more symptomatic) SRS-2 and BRIEF2 scores. For Autism Spectrum, the participants in the ‘Above Average’ group also showed higher SSiS Problem Behaviors and lower EVT-2/3 scores. Finally, the participants in the ‘Above Average’ group for Hyperactivity/Inattention and Externalizing were also older in age.

## 4. Discussion

This study examined the SSiS in a large sample of youth with DS. The average social skills standard score across the sample was 92, falling well within 1 standard deviation from the mean of the normative sample. Similarly, the average problem behaviors standard score was 104. Upon examining behavior levels by subscale, most of the sample fell within the average range for each subscale. Thus, as a whole, youth with DS showed relatively mild symptoms associated with social interaction skills measured by the SSiS. This was surprising and conflicts with Guralnick et al.’s (2009, 2011) findings of significantly lower scores on the earlier version of this instrument, the SSRS, in 4- to 6-year-old children with DS. It also conflicts with the idea that as children with DS age and the demands of social interaction increase, they fall further behind their peers, especially in higher-order skills such as social cognition, social reasoning, and social problem-solving. However, the SSiS does not measure these individual skills but rather relies on informant report about behaviors that result from the coordination of many different social skills in real-world settings. Thus, it is possible that older children with DS develop compensatory strategies for navigating social interactions in naturalistic settings where more social cues are available. Regardless, informant report measures such as the SSiS are important to consider clinically to gather meaningful information about social functioning across settings.

The current study provides some of the first evidence validating the use of the SSiS in youth with DS. SSiS composite scores correlated in the expected directions with SRS-2, BRIEF2, and EVT-2/3 composite scores of autism characteristics, executive function, and expressive vocabulary, respectively. Moreover, there was differentiation in the strength of these correlations such that EVT-2/3 expressive vocabulary was significantly and moderately correlated with SSiS social skills but only weakly correlated (failing to reach significance) with SSiS problem behaviors. Additionally, BRIEF2 executive function impairments were strongly correlated with SSiS problem behaviors and moderately correlated with SSiS social skills. Further, differentiation in the strength of associations between the SSiS subscales and subdomain/index scores on the SRS-2 and BRIEF2 provide initial evidence of construct validity, although this should be systematically tested through future research.

Across the sample, participants showed different distributions of low/average/high scores by subscale. That is, for some subscales, a disproportionate amount of the sample scored in the below average (i.e., lower levels of social skills) or above average (i.e., more symptomatic in problem behaviors or autism spectrum) range relative to the normative sample; for other subscales, the sample’s score distribution aligned more closely to that of the normative sample. For social skills, the subdomain with the greatest proportion of the sample scoring in the below average range was *Assertion*. These items refer to skills such as initiating a conversation, asking for help, and speaking up for oneself. The next subscale with a disproportionate amount of the sample in the below average range was *Responsibility*, which includes items related to taking responsibility for one’s own actions, following through, and showing respect for others’ property. The next subscale in the below average range was *Engagement*, which includes initiating and joining interactions with peers, making friends, and starting conversations with others. The final social skills subscale that showed only slightly more children in the below average range relative to the normative sample was *Cooperation*, which mostly asks about the home environment—following rules or caregiver instructions and getting along at home. In contrast, the subscales for which the sample with DS more closely aligned with the normative sample were *Empathy*, which includes showing concern for others and trying to understand their feelings, *Communication*, which includes mostly nonverbal pragmatic skills like turn-taking, appropriate tone and eye contact, and gesturing, as well as *Self-Control*, which includes staying calm when others are aggressive or disagree and compromising.

Aspects of this profile fit broadly with what is known about the DS phenotype. For example, during experimental or language sampling tasks, children with DS have initiated conversational topics and signaled their own non- comprehension to repair conversational breakdowns less often than developmental age-matched typically developing peers (see Martin et al., 2009 for a review; Martin et al., 2018). This aligns with the lower scores observed for *Assertion* and *Engagement* in the current study. The findings for *Empathy* and *Communication* in the current sample align with observations of relatively strong empathic and pro-social behaviors noted by Kasari et al. (2003) and relative strengths in nonverbal communication noted in studies of early social development in young children with DS (see Cebula et al., 2010 for a review). The current study extends prior research by characterizing a profile of relatively more and less impaired social skills used during everyday social interaction by children and adolescents with DS.

For problem behaviors, the subdomain with the highest proportion of the sample scoring in the above average range was *Hyperactivity/Inattention*. These items refer to impulsive behaviors and interrupting, fidgeting, distractibility, and temper tantrums. Slightly more children scored in the above average range for *Externalizing*; interestingly, many of the *Hyperactivity/Inattention* subscale items feed into this subscale, but it also includes unique items related to disobedience and defiance. The subscale scores for which the sample with DS more closely aligned with the normative sample were *Bullying* and *Internalizing*. These findings correspond with other research pointing to particularly high rates of hyperactivity, inattention, and noncompliance among youth with DS (Capone et al., 2006; Jacola et al., 2014; Patel et al., 2018; Esbensen et al., 2021) along with slightly elevated aggressive behaviors (van Gameren-Oosterom et al., 2011) and relatively low rates of internalizing symptoms, at least during childhood (van Gameren-Oosterom et al., 2011, 2013; Channell et al., 2019). Interestingly, both *Hyperactivity/Inattention* and *Externalizing* scores were negatively correlated with age, indicating a decrease in symptoms across age in the current sample. Broadly speaking, this fits with others’ reports of age-related differences in the pattern of maladaptive behaviors in DS across childhood and adolescence (Dykens, 2007; van Gameren-Oosterom et al., 2011, 2013).

Finally, 31% of the sample scored in the above average range for the *Autism Spectrum* subscale, which includes both social skills and problem behaviors commonly associated with features of autism. This is largely consistent with other findings that children with DS tend to show elevated scores on autism screeners and symptom monitoring measures that capture broad autism-like characteristics (DiGuiseppi et al., 2010; Warner et al., 2014; Channell et al., 2015; Channell, 2020).

It is also important to recognize the variability observed across this study’s sample in SSiS composite standard scores and in the distribution of scores across subscales reported in Figures 1 and 2. That is, a range of scores were observed for the SSiS social skills and problem behaviors composites and in the distribution of scores across subscales. Additionally, we plotted participants’ sores on the other study measures of autism characteristics, executive function, and expressive vocabulary, with separate plots for the subsample of participants who scored in the below/above average (social skills/problem behaviors) on a subscale relative to the remainder of the sample (see Figures 3, 4). These data reveal systematic differences among subsamples of participants who show greater impairments vs. those who show average or better social skills and problem behaviors. These results support Channell et al.’s (2021) findings of potential subgroups within the DS phenotype. However, the current study did not use latent profile analysis, and the extent to which the same participants who fell in the below/above average category across the different subscales is unknown. Regardless, these data demonstrate the importance of considering individual differences within the DS phenotype (Karmiloff-Smith et al., 2016).

### 4.1. Limitations and future directions

Although the current sample size was large, the study did not employ an epidemiological design. Therefore, one should not interpret the SSiS mean composite scores or percentages of children falling into the behavioral levels as such. More work is needed to determine the extent to which these findings generalize (or do not generalize) to the larger population with DS. Based on other measures included in this study, the current sample appears largely similar to what is reported in the literature about DS in terms of IQ (see Grieco et al., 2015), executive function (e.g., Loveall et al., 2017; Rosser et al., 2018), and autism characteristics (Channell et al., 2015); however, convenience sampling is a common issue in DS research. Additionally, the current sample was 84% White and 94% Non-Hispanic, much less diverse than recent United States population-based data on DS reported by Mai et al. (2019; 62% Non-Hispanic and 43% White Non-Hispanic) and United States Census estimates (76% White and 81% Non-Hispanic; U.S. Census Bureau, 2022), although race and ethnicity were categorized differently in these reports than in the current study. Thus, it will be important to examine the utility of the SSiS across more culturally diverse samples in which cultural expectations for social interaction may also differ. Furthermore, we combined data from two different studies to yield a larger sample size; however, both studies used different inclusion/exclusion criteria. Notably, the UIUC study required that participants were able to speak in phrases and use spoken language as their primary mode of communication, who may also be more advanced developmentally. Thus, it is possible that we had an overrepresentation of individuals with DS with more advanced skills in the current sample. Further, both studies required that participants could complete an in-person assessment battery. This criterion likely led to the exclusion of children with DS with more maladaptive behaviors and/or lower developmental levels from these studies. Such children may have more limited social interaction skills or a different social skill profile than what was observed in the current sample, again limiting generalizability to the broader population with DS. Future research should focus on examining the SSiS in a more inclusive sample of children with DS, both developmentally and demographically.

### 4.2. Conclusion

Very few measures of social skills have been established for youth with DS, particularly for older youth who experience increased social demands. The SSiS is a social skills assessment tool that is useful clinically because it provides information about how an individual functions during day-to-day social interactions across different settings. The results provide preliminary data on the SSiS in youth with DS and contribute some of the first evidence validating its use in this population. This study also informs the understanding of the DS phenotype by using the SSiS to report social skills profiles across a sample of children and adolescents with DS in relation to autism characteristics, executive function, and expressive language. Moving forward, a well-researched tool for measuring everyday social interaction skills in this population will advance the understanding of this aspect of the DS phenotype and will provide clinicians with an assessment tool for diagnosing, intervention planning, and treatment monitoring.

## Data availability statement

The raw data supporting the conclusions of this article will be made available by the authors, without undue reservation.

## Ethics statement

The studies involving human participants were reviewed and approved by the University of Illinois Urbana-Champaign, Cincinnati Children’s Hospital Medical Center, and Colorado State University. Written informed consent to participate in this study was provided by the participants’ legal guardian/next of kin.

## Author contributions

MC, LM, and AE conceptualized this study. MC, DF, and AE led the original studies from which data were used in this study. MC led manuscript drafting and conducted data analysis. MC, LM, ES, DF, and AE edited the manuscript. All authors contributed to the article and approved the submitted version.

## Funding

This research was supported by the *Eunice Kennedy Shriver* National Institute of Child Health and Human Development of the National Institutes of Health through grants R03HD083596 (PI: MC), R01HD093754 (PI: AE), R01HD099150-0 (PI: DF), T32HD007489, and P50HD105353.

## Conflict of interest

The authors declare that the research was conducted in the absence of any commercial or financial relationships that could be construed as a potential conflict of interest.

## Publisher’s note

All claims expressed in this article are solely those of the authors and do not necessarily represent those of their affiliated organizations, or those of the publisher, the editors and the reviewers. Any product that may be evaluated in this article, or claim that may be made by its manufacturer, is not guaranteed or endorsed by the publisher.
